# Characterization of Fractional Polysaccharides from *Gleditsia sinensis* and *Gleditsia microphylla* Gums

**DOI:** 10.3390/molecules21121745

**Published:** 2016-12-19

**Authors:** Yantao Liu, Zhenglong Xu, Weian Zhang, Jiufang Duan, Jianxin Jiang, Dafeng Sun

**Affiliations:** 1 MOE Engineering Research Center of Forestry Biomass Materials and Bioenergy, Beijing Forestry University, Beijing 100083, China; liuyt0321@163.com (Y.L.); zwa728@sina.com (Z.X.); ziyuanbeijing@163.com (W.Z.); duanjiu99@163.com (J.D.); 2 Nanjing Institute for the Comprehensive Utilization of Wild Plant, Nanjing 210042, China

**Keywords:** *Gleditsia sinensis*, *Gleditsia microphylla*, ethanol, isopropanol, fractionation

## Abstract

The seeds of *Gleditsia sinensis* and *Gleditsia microphylla*, widespread in China, are an important source of galactomannans. *G. sinensis* gum (GSG) and *G. microphylla* gum (GMG) were purified and precipitated using different concentrations of ethanol and isopropanol. The GSG and GMG, precipitated in different stages, presented different characteristics, including polymer recovery, mannose/galactose ratio, chemical composition, molecular weight, and morphological appearance. The galactomannan recovery of GSG and GMG in 33.3% ethanol was 81.7% and 82.5%, respectively, while that in 28.8% isopropanol was 81.3% and 82.9%, respectively. To achieve similar precipitation efficiency, the amount of isopropanol should be lower than that of ethanol because of the lower dielectric constant of isopropanol (20 vs. 25 for ethanol). The precipitation behavior of galactomannans in polar organic solvents was dependent on the molecular structures and properties of the solvent. A higher mannose/galactose ratio and a higher molecular weight was obtained in a lower concentration of alcohols.

## 1. Introduction

Galactomannans are found in the endosperm of numerous plants, particularly the Leguminosae. Galactomannan gums are high-molecular-weight carbohydrates comprising a β-(1,4)-d-mannan backbone with single α-(1,6)-linked d-galactose branches. Their mannose/galactose (M/G) ratios and molecular mass distribution differ according to species [1].

Galactomannans can often be used in different forms for human consumption. They are excellent stiffeners and stabilizers of emulsions, and have wide applications in various industries, including the textile, pharmaceutical, biomedical, cosmetics, and food industries. However, most galactomannans used in pharmaceutical technology and cosmetics are usually unpurified gums [2,3].

Recently, alternative sources of seed gums have attracted increasing attention. The seeds of *G. sinensis* and *G. microphylla* (Leguminosae family) are two nontraditional sources of galactomannans. *G. sinensis* and *G. microphylla* are woody species that can grow in a wide range of environmental conditions. In China, they are widely distributed and are used as food, medicine, and health care products, among others [4,5,6].

Purification of these polymers can eliminate impurities and endogenous enzymes in coarse samples, improving their mass stability in the application process. Using ethanol and isopropanol [7,8] as solvents to obtain these carbohydrates can promote intramolecular associations between water-soluble polymers through water competition. The dielectric constants of ethanol (20) and isopropanol (25) are much lower than that of water (80) at 25 °C [9]. Therefore, adding ethanol and isopropanol can reduce the dielectric constant of the polysaccharide solution and induce conformational changes in the polymer, thus allowing molecules to aggregate and precipitate.

In many studies, alcohols and other organic solvents have been used to precipitate galactomannans or xyloglucan [10] from the water-extracted gum solution [7,11,12,13]. However, most of these studies have focused on one-time purification with high alcohol concentrations; only a few studies have focused on fractional purification and precipitate characterization of the polysaccharide gum. Precipitation is important for the purification and isolation of galactomannan gum; however, little research has been conducted on the relationship between the molecular structure of the polymer and its precipitation behavior in alcohol.

In the present work, two types of galactomannans (*G. sinensis* gum (GSG) and *G. microphylla* gum (GMG)) with different molecular mass distributions and M/G ratios were fractionally precipitated in ethanol– and isopropanol–water solutions. Other studies have used solvents such as chloroform [14] and petroleum ether [15] to extract galactomannans, which are not authorized in the food industry. The characterizations of the galactomannan precipitates, as well as their M/G ratios, monosaccharide composition, intrinsic viscosity, molecular mass distribution, ash content, and polymer recovery yields, were determined, thus providing useful information about the purification of galactomannan gums and the influence of the molecular structure on their behavior.

## 2. Results and Discussion

### 2.1. Chemical Composition and Rheological Properties

The chemical composition of the crude and purified gums GSG and GMG are listed in Table 1. The effect of the chemical composition on the crude and purified gums with different solvents and its concentration was significant (*p* < 0.05). The galactomannan contents of the crude GSG and GMG were 86.3% and 71.5% on the dry mass basis, respectively. The M/G ratios obtained for crude GSG and GMG were 3.0 and 3.2, respectively, which are consistent with the previous literature values [16]. They were very similar to the commercial tara gum (3.0), which is a kind of galactomannan gum and is widely used as a thickening agent and stabilizer in food applications [17]. The protein and ash contents were found to decrease dramatically after purification by ethanol and isopropanol precipitation. This phenomenon was in agreement with reports by other researchers [18]. In addition, for both gums, the purity of the sample precipitated by isopropanol was a little higher than that obtained by ethanol.

**Figure 1:**
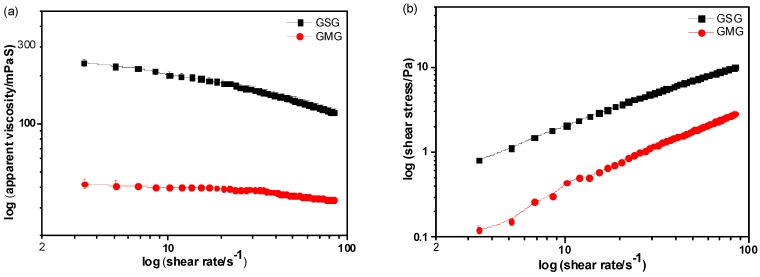
Flow curves of 0.5% (*w*/*w*) aqueous solutions of GSG (**a**) and GMG (**b**) at 30 °C.

The flow curves of the 0.5% (*w*/*w*) aqueous solutions of GSG and GMG are shown in Figure 1. The apparent viscosity of the gum solution decreased and the shear stress increased with the increasing shear rate, indicating a typical non-Newtonian flow for the polymer [19,20]. For a solution to be considered Newtonian, its flow behavior index (*n*) must be equal to 1, while a value of 1 > *n* > 0 indicates a pseudoplastic fluid behavior [21,22]. As shown in Table 2, the *n* values of all the gum solutions were less than 1.00, suggesting that they were non-Newtonian fluids. The *n* values of the 0.5% GSG and GMG solutions were 0.760 and 0.963, respectively, which also suggests that the two galactomannan-type gums have a shear-thinning behavior, usually assigned to the pseudoplastic fluids [23,24]. The characteristic of the pseudoplastic fluid of galactomannan from GSG and GMG might be due to the entanglement interactions between the polymer chains. Therefore, at a lower shear rate, the longer molecular chains of GSG presented a random-coil conformation in aqueous solutions and had a correspondingly higher apparent viscosity, while, at a higher shear rate, the successively increasing shear stress could lead to the deformation or breakdown of intermolecular linkages and therefore a decline in the apparent viscosity was observed [25]. The *n*-values of guar gum (GG) and locust bean gum (LBG) solution were 0.571 and 0.834 [26], respectively, which means that GSG and LBG solutions are similar, implying that the GSG and LBG have similar shear stress and apparent viscosity. However, the *n*-value of the GMG solution was 0.963, which was much higher than that of GSG, LBG, and GG. This difference of the rheological behavior of GSG and GMG may be because the galactomannan content and the molecular weight (from Table 2) of the crude polymer from *G. microphylla* was much lower than that of the crude from *G. sinensis*.

### 2.2. Galactomannan Recovery and M/G Ratio of the Precipitated Fractions

**Figure 2:**
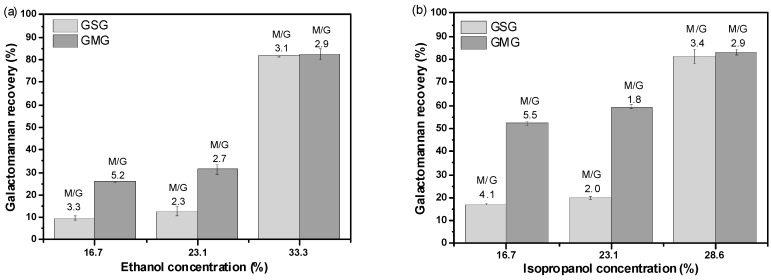
Galactomannan recovery yield and M/G ratio of the gum fractions of GSG and GMG precipitated by (**a**) ethanol and (**b**) isopropanol. Numbers over the bars represent the M/G ratios.

The recovery yields and M/G ratio of the galactomannan fractions for both polymers obtained by gradual alcohol precipitation are displayed in Figure 2; the calculation of the recovery yield (Equations (2)–(4), Section 3.2.4) was based on the galactomannan amount in the original crude gum dispersion. The total galactomannan recovery yields of GSG and GMG by ethanol precipitation (Figure 2a) were 81.7% and 82.5%, while those obtained by isopropanol precipitation (Figure 2b) were 81.3% and 82.9%, respectively, which do not seem to be statistically different. Even though it was evident that, to achieve a similar precipitation efficiency, the amount of ethanol used should be higher than that of isopropanol. The difference can be attributed to the lower dielectric constant of isopropanol. It can also be observed from Figure 2 that, during the fractionation process of both the ethanol and isopropanol precipitations, the recovery yield of galactomannan from GMG was always higher than that from GSG, except for the higher concentrations of the alcohols used. Perhaps the chemical differences between the two gums (GSG and GMG) can cause some changes during precipitation. One possible interpretation of these results is that, because the galactomannan content of crude GMG was lower than that of crude GSG, this allowed the galactomannan of GMG to precipitate more easily and promptly in the same concentration of both alcohols. It is well known that the solution properties of galactomannans are greatly affected by the degree and pattern of galactose substitution in galactomannan and that the solubility of galactomannans are directly related with the increasing H-bonding density provided by the galactose side groups [27]. As shown in Figure 2, the M/G ratio obtained during the first stage of precipitation was higher than that of the corresponding crude gum (Table 1), the precipitation with the high molecular weight (Table 3), and the high value of M/G formed at low alcohol concentration. This might be explained by the fact that low-molecular-weight and highly substituted components are not as easily precipitated by ethanol or isopropanol as other solvents [28]. In another study, with the precipitation carried out in three stages, the molecular weight of the LBG decreased while its M/G ratio increased with increasing alcohol concentration [26]. From Figure 2 and Table 3, we can see the precipitates of GSG and GMG formed in the first stage had the highest molecular weight (Table 3) and the highest M/G ratio. The high molecular weight of both polymers obtained from GSG and GMG were less easily purified and precipitated in a low alcohol concentration.

### 2.3. Fractional Precipitation by Ethanol and Isopropanol

**Figure 3:**
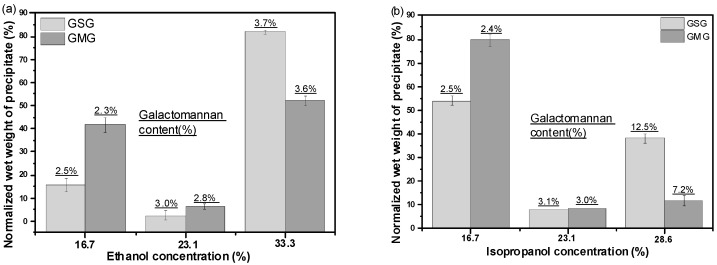
Normalized wet mass ratio and galactomannan concentration of the gum fractions of GSG and GMG obtained from (**a**) ethanol and (**b**) isopropanol precipitation.

The wet weights of the three precipitated fractions of each gum was normalized to 100% (Equation (3), Section 3.2.4). The wet mass ratio and galactomannan concentration of each gel-like precipitate are summarized in Figure 3. In an ethanol solution (Figure 3a), the precipitates obtained with the highest alcohol concentration of the fractional precipitation procedure represented the largest proportion of the total precipitates; and, the similar results also obtained in the isopropanol solution (Figure 3b). With the increase in alcohol concentration in the bulk solution, the galactomannan content of the separated gel-like precipitate gradually increased, probably because of the increasing compactness of the precipitate [26]. The galactomannan concentration in the isopropanol-induced precipitate was significantly higher than that in the ethanol-induced precipitate because of the lower dielectric constant of isopropanol, resulting in a more compact precipitate than that found in ethanol. It can be concluded from Figure 3 that the high molecular weight (Table 3) and low galactomannan concentration of the precipitates formed at low ethanol/isopropanol concentrations were owing to the high levels of the entrained solvent in the gel-like precipitates; a further increase in the ethanol/isopropanol content increased the galactomannan concentration and purity [18,28]. At the same time, the morphological appearance of the ethanol/isopropanol precipitation had been observed in the first and third stages. In the first stage, precipitation from both alcohols formed a crude gum with a yellow, loose appearance; in the third stage, the alcohol/isopropanol precipitation formed a pure gum with a white, compact appearance. After the alcohol precipitation, especially the higher concentration of alcohol, the polysaccharide showed a marked increas in the purity with a bright whitish color.

### 2.4. Molecular Weight

The changes in the molecular-weight distribution in the ethanol and isopropanol fractionation process for GSG and GMG are shown in Table 3. It can be seen from Table 3 that the M_w_ values for crude gum of GSG and GMG were 7.865 × 10^5^ and 6.245 × 10^5^ Da, respectively. This result of the M_w_ values was consistent with the rheological properties of our previous measurements of the crude gum of GSG and GMG (as shown in Figure 1). After extracted with hot water of the crude gums, at lower alcohol concentrations, the molecular weight of the precipitated galactomannans was higher than those of the corresponding crude gums, indicating that the component of higher molecular weight was more prone to precipitation than those of lower molecular weight. This phenomenon may be due to the fact that the conformations of galactomannan fractions with different molecular weights might transform in water–alcohol solutions. Specifically, in an aqueous solution, polysaccharide molecules connect with water molecules through hydrogen bonds to maintain the solubility of the polysaccharide in water. Moreover, the addition of polar organic solvents such as ethanol and isopropanol would strengthen the intramolecular hydrogen bonding and induce conformational change and aggregation of polysaccharide molecules, eventually resulting in the precipitation of the molecule [29]. Consequently, adding ethanol or isopropanol to an aqueous solution of polysaccharide initially to obtain a certain concentration alcohol solution resulted in the precipitation of macromolecular chains, and with the increasing of alcohol added, the relatively low-molecular-weight fragments would precipitate, thus realizing the aim of fractional precipitation of polysaccharides on the basis of molecular structure. Therefore, it can be concluded that the conformational transition of galactomannans in polar ethanol and isopropanol is affected by the molecular structure (such as galactose substitution and molecular weight), and especially by inter- and intra-molecular hydrogen bonding.

## 3. Materials and Methods

### 3.1. Materials

*G. sinensis* and *G. microphylla* seeds were supplied by Shexian Forestry Bureau in Hebei, China. The samples were collected in December 2012. They were manually separated and kept in a cool and dry place for further use. Moisture content of the whole seed was dried at (105 ± 2 °C) for constant weight (10.0% (*w*/*w*)). The standard monosaccharides (l-rhamnose, l-arabinose, d-glucose, d-galactose, d-mannose, and d-xylose) were purchased from Sigma-Aldrich (Saint Quentin Fallavier, France). Dextran standards (DXT3k, DXT25k, DXT160k, DXT760k, and DXT1185k) with an average molecular weight range of 3.7 × 10^3^~1.2 × 10^6^ Da were purchased from TosoHaas (Tokyo, Japan). Absolute ethanol and isopropanol were purchased from Beijing Chemical Works (Beijing, China). Calcium carbonate and concentrated sulfuric acid were all purchased from China National Pharmaceutical Group Corporation (Shanghai, China). Syringe filters were supplied by Tianjin branch billion Lung Experiment Equipment Co. Ltd., (Tianjin, China).

### 3.2. Methods

#### 3.2.1. Dehulling Pretreatment and Gum Preparation

Baking treatment was used in dehulling pretreatment. About 30 g of seeds were put into the microwave oven (Galanz, G80F23DCN3L-F7(R0)) and baked for 6 min (just only use light wave mode 800 W), followed by crushing during 3 s in a laboratory muller. After baking treatment, the hull was easily crushed into fragments, while the endosperm was harder crushing and almost unbroken in the crush. Most of the endosperm fragments were separated by sieving, the rest were manually separated. The endosperm was subsequently milled through a sieve size of 0.125 mm. The two crude gums of GSG and GMG were obtained and subsequently were stored in a dryer for further use.

#### 3.2.2. Rheological Properties

The rheological properties of the crude gums obtained from the seeds of *G. sinensis* and *G. microphylla* were carried out in a LVDV-III Ultra Rheometer (Brookfield Engineering Laboratories, Stoughton, MA, USA) equipped with a small sample adapter (SC13R) and spindle SC4-31. Samples for rheological measurement were prepared at a gum concentration of 0.5% (*w*/*w*) in distilled water on the dry weight basis. Then, the prepared gum solutions were placed in a water bath at 80 °C for 30 min under magnetic stirring to produce uniform dispersion and complete hydration. The apparent viscosity of these crude gum solutions at 0.5% (*w*/*w*) were determined at 30 °C using exactly 9.0 mL of solution. All measurements were conducted at least in triplicate. Curves of the shear stress (σ) as a function of the shear rate (γ) for the two gum solutions were obtained by the software (Rheocalc V3.2, Stoughton, MA, USA) with the shear rate increasing from 1.7 to 85.0 s^−1^ within 4.5 min.

σ = k × γ^n^ (1)

where k is the consistency index, and n is the flow index.

#### 3.2.3. Fractionation of the Crude Gums

**Figure 4:**
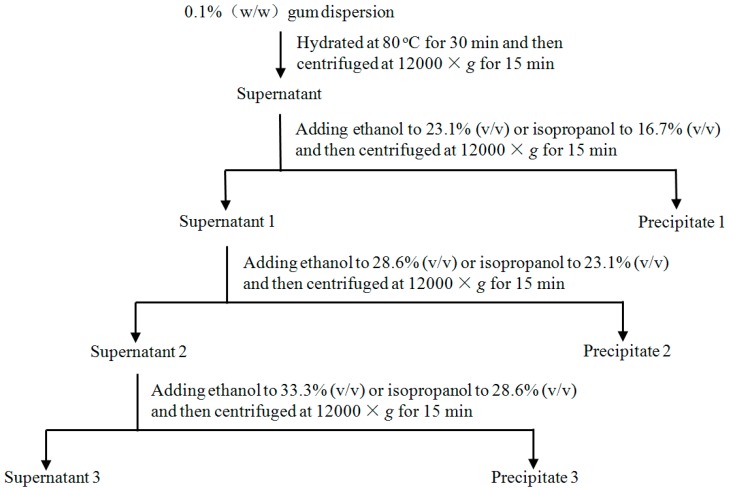
Scheme of the fractional precipitation of the two galactomannan gums of with ethanol and isopropanol.

Two fractions for each of the gums from *G. sinensis* and *G. microphylla* were precipitated by the gradual addition of ethanol and isopropanol. First, however, the crude gum was dispersed into distilled water to achieve a gum concentration of 0.1% (*w*/*w*), and was then placed in a water bath at 80 °C for 30 min with mechanical stirring. After full hydration, the gum dispersion was centrifuged at 12,000× *g* for 15 min to remove the insoluble residues, and the supernatant was collected for further fractional precipitation with ethanol and isopropanol. A certain volume of ethanol or isopropanol was added slowly to the supernatant under constant stirring to obtain a precipitate at room temperature (25 ± 3 °C). The resulting solution was centrifuged at 12,000× *g* for 15 min to separate the precipitate. The operation was repeated until no obvious precipitation was observed. All precipitates with the same concentration of alcohol were joined together. The wet precipitate was weighed and then dried in a vacuum oven for 6 h at 60 °C for constant weight [10]. The detailed fractionation procedure is summarized in Figure 4.

During the fractional precipitation of the polysaccharides, the alcohol concentration in the alcohol–water solution was stepwise increased, and the formed polysaccharides precipitates were collected in each step, according to the method used by [26]. The precipitation experiment was carried out at least in triplicate.

#### 3.2.4. Chemical Composition of the Crude and Purified GSG and GMG 

The protein contents of *G. sinensis* and *G. microphylla* gums were performed according to the Kjeldahl method [30], after mineralization and distillation, and determined using a conversion factor of 6.25 [31]. The ash content of the gums was determined gravimetrically after dry mineralization at 550 °C for 5 h [31]. The galactomannan value of the gum samples was calculated, through the sum of mannose and galactose contents determined by HPLC monosaccharide analysis [7]. All the measurements were conducted at least in triplicate.

After a two-step hydrolysis with sulfuric acid, monosaccharide composition of the polysaccharide fraction was performed according to the NREL laboratory analytical procedure method [32]. In summary, the carbohydrate fraction was determined by hydrolyzing the gum sample (300 mg) with 3 mL of 72% (*w*/*w*) H_2_SO_4_ for 1 h at 30 °C in a pressure tube with occasional vibration. Then, 84 mL of distilled water was added into the tube to achieve an acid concentration of 4.0% (*w*/*w*), and the sample was autoclaved for 1 h at 121 °C. After acid hydrolysis, the sample was neutralized with calcium carbonate. Hydrolyzed samples were filtered through a 0.22 μm filter (Tianjin branch billion Lung Experiment Equipment Co. Ltd., Tianjin, China) and injected to a Waters 2695 HPLC system equipped with an Aminex HPX-87P column (300 mm × 7.8 mm, Bio-Rad, Hercules, MA, USA) at 85 °C and refractive index detector (2414 RID; Waters, Milford, MA, USA) at 35 °C. Double distilled water was used as the mobile phase with a flow rate of 0.6 mL/min. Calibration was performed with a series of standard sugar solutions of l-rhamnose, l-arabinose, d-glucose, d-galactose, d-mannose, and d-xylose.

The galactomannan recovery yield, the normalized weight percentage, and the polysaccharide content were calculated according to the following Equations:

(2) $$Recovery\;\left(\%\right)=\frac{\sum_{i=1}^{n}Galactomannan\;amount\;of\;Fraction\;i\;\left(mg\right)}{Galactomannan\;amount\;of\;crude\;gum\;\left(mg\right)}\times 100$$

(3) $$Normalized\;weight\;percent\left(\%\right)=\frac{wet\;weight\;of\;Fraction\;i\;\left(mg\right)}{total\;wet\;weight\;of\;all\;Fractions\;\left(mg\right)}\times 100$$

(4) $$Content\;\left(\%\right)=\frac{Galactomannan\;amount\;of\;Fraction\;i\;\left(mg\right)}{wet\;weight\;of\;Fraction\;i\;\left(mg\right)}\times 100$$

#### 3.2.5. Molecular Mass Determination

The weight-average molecular weights (M_w_) of *G. sinensis* and *G. microphylla* gums were determined by gel permeation chromatography (GPC) equipped with a refractive index detector and three columns in series, TSK PW_XL_ guard column, TSK_gel_ G 6000 PW_XL_ and TSK_gel_ G 3000 PW_XL_ (TosoHaas, Tokyo, Japan). The mobile phase was a 0.2 M phosphate buffer at pH 6.8, with a flow rate of 0.6 mL/min. Samples were diluted to approximately 1.0 g/L and filtered through a 0.45 mm filter (Tianjin branch billion Lung Experiment Equipment Co. Ltd. (Tianjin, China)) prior to injection onto the column. Dextran standards, with average molecular weights of 3.7 × 10^3^~1.2 × 10^6^ Da, were used to calibrate the columns, which were operated at 35 °C during measurements. The weight-average molecular weight (M_w_), the number-average molecular weight (M_n_), and M_w_/M_n_ were calculated with the Empower 2 software (Waters, Milford, MA, USA).

### 3.3. Statistical Analysis

The statistical significance of the experimental data was examined by analysis of variance (ANOVA) using SPSS version 17.0 (SPSS Inc., Armonk, NY, USA). The *p*-values were used as a tool to check the significant difference of the chemical compositions of the crude and purified gum. Values of *p* less than 0.05 (*p* < 0.05) indicate a significant difference. A smaller *p*-value illustrates that the significance of the variable is more obvious.

## 4. Conclusions

GSG and GMG could be fractionated through gradual precipitation in ethanol and isopropanol. The purity of galactomannans precipitated by the ethanol and isopropanol precipitation was improved significantly and the property of a pseudoplastic solution was also confirmed in these two galactomannans. At low concentrations of alcohol, the formed precipitates were found to have a high mass ratio and low galactomannan concentration because of the high levels of the residual solvent. In addition, the concentration and purity of galactomannans increased with increasing alcohol concentration. To achieve the same precipitation efficiency, the amount of ethanol should be higher than that of isopropanol because of the low dielectric constant of isopropanol. In polar organic solvents, the precipitation behavior of galactomannans was related to the molecular structure such as galactose substitution and molecular weight, which was also closely linked to the aqueous solubility (hydration) of galactomannan.

## Figures and Tables

**Table 1:** Chemical composition of the crude and purified *G. sinensis* gum (GSG) and *G. microphylla* gum (GMG), both in ethanol and isopropanol.

Samples	Crude Gum	Ethanol-Purified GSG ^2^	Isopropanol-Purified GSG ^3^	*p*-Value	Crude GMG	Ethanol-Purified GSG ^2^	Isopropanol-Purified GSG ^3^	*p*-Value
Protein (%)	4.00 ± 0.02 ^1^	0.96 ± 0.03	0.94 ± 0.02	<0.001	7.41 ± 0.06	0.86 ± 0.02	0.76 ± 0.02	<0.001
Ash (%)	0.74 ± 0.09	0.32 ± 0.02	0.20 ± 0.02	0.012	1.54 ± 0.05	0.38 ± 0.03	0.28 ± 0.02	<0.001
Galacto-Mannan (%)	86.3 ± 0.2	94.0 ± 0.8	95.3 ± 0.7	0.004	71.5 ± 0.4	88.2 ± 0.3	89.6 ± 0.4	<0.001
M/G ^4^	3.0 ± 0.03	3.1 ± 0.07	3.4 ± 0.06	0.031	3.2 ± 0.004	2.9 ± 0.07	2.9 ± 0.06	0.044

^1^ Data are mean ± SD of three determinations on a dry weight basis. ^2^ Galactomannan gum purified by 33.3% ethanol precipitation. ^3^ Galactomannan gum purified by 28.6% isopropanol precipitation. ^4^ M/G: mannose/galactose ratio.

**Table 2:** Rheological parameters of the crude GSG, GMG, GG, and LBG.

Power-Law Model	*k*	*n*	*R*^2^	References
Crude GSG	0.35	0.760	0.997	
Crude GMG	0.04	0.963	0.994	
Crude GG ^1^	0.99	0.571	0.985	[26]
Crude LBG ^2^	0.27	0.834	0.998	[26]

^1^ GG here means guar gum and ^2^ LBG means locust bean gum. *R^2^* means correlation with the fitting equation (Equation (1), Section 3.2.2).

**Table 3:** The M_w_ M_n_ and M_w_/M_n_ of GSG and GMG during the ethanol/isopropanol fractional precipitation procedure.

Samples	GSG			GMG		
	M_w_/Da	M_n_/Da	M_w_/M_n_	M_w_/Da	M_n_/Da	M_w_/M_n_
Crude gum	7.865 × 10^5^	5.042 × 10^5^	1.56	6.245 × 10^5^	5.027 × 10^5^	1.24
23.1% Ethanol	2.925 × 10^6^	2.120 × 10^6^	1.38	2.958 × 10^6^	2.447 × 10^6^	1.21
28.6% Ethanol	3.466 × 10^5^	2.611 × 10^5^	1.33	4.007 × 10^5^	3.710 × 10^5^	1.08
33.3% Ethanol	1.891 × 10^5^	1.482 × 10^5^	1.28	2.116 × 10^5^	2.050 × 10^5^	1.03
16.7% Isopropanol	3.177 × 10^6^	2.075 × 10^6^	1.53	3.139 × 10^6^	3.031 × 10^6^	1.04
23.1% Isopropanol	3.635 × 10^5^	3.537 × 10^5^	1.03	4.135 × 10^5^	3.542 × 10^5^	1.17
28.6% Isopropanol	5.816 × 10^5^	3.853 × 10^5^	1.51	7.707 × 10^5^	4.271 × 10^5^	1.80
